# Rapid Functional Drug Screening of Passage-Zero Patient Brain Tumor Tissues Ex Vivo: Results from a Clinical Feasibility Study

**DOI:** 10.21203/rs.3.rs-7993619/v1

**Published:** 2025-11-04

**Authors:** Andrew Satterlee, Breanna Mann, Nichole Artz, Adebimpe Adefolaju, Alain Valdivia, Xiaopei Zhang, Rajaneekar Dasari, Caroline Stockwell, Morrent Thang, Allison Murray, Noah Bell, Andrew Buckley, Rami Darawsheh, Kerry Fitzgerald, Jonathan Williams, Hardik Parikh, Shuang Gao, Jie An, Yvette Rodriguez, Daniel Metzger, Collin Parrow, Dylan Riley, Robert Seager, Stephanie Hastings, Taylor Jensen, Shakti Ramkissoon, Dominique Higgins, Yasmeen Rauf, Scott Elton, Kimberly Hamilton, Jeremy Wang, Albert Baldwin, Shawn Hingtgen, David Kram

**Affiliations:** University of North Carolina at Chapel Hill; University of North Carolina at Chapel Hill; University of North Carolina at Chapel Hill; University of North Carolina at Chapel Hill; University of North Carolina at Chapel Hill; University of North Carolina at Chapel Hill; University of North Carolina at Chapel Hill; University of North Carolina at Chapel Hill; University of North Carolina at Chapel Hill; University of North Carolina at Chapel Hill; University of North Carolina at Chapel Hill; University of North Carolina at Chapel Hill; University of North Carolina at Chapel Hill; Labcorp; Labcorp; Labcorp; Labcorp; Labcorp; Labcorp; Labcorp; Labcorp; Labcorp; Labcorp; Labcorp; Labcorp; Labcorp Oncology; University of North Carolina at Chapel Hill; University of North Carolina at Chapel Hill; University of North Carolina at Chapel Hill; University of North Carolina at Chapel Hill; University of North Carolina at Chapel Hill; University of North Carolina at Chapel Hill; University of North Carolina at Chapel Hill; University of North Carolina at Chapel Hill

## Abstract

Functional precision medicine (FPM) offers a promising path forward in neuro-oncology, where genomic profiling alone often fails to predict therapeutic response. To bridge this gap, we developed the Screening Live Cancer Explants (SLiCE) platform, a rapid *ex vivo* drug screening assay that functionally tests passage-zero patient brain tumor tissues engrafted atop living organotypic brain slice cultures (OBSCs). With an assay time of just four days, SLiCE preserves key tumor characteristics not maintained *in vitro*, including genomic fidelity, growth, invasion, and treatment response, with higher engraftment rates and faster assay speeds than *in vivo* models. Our standard cryopreservation workflow enables reproducible, iterative, and on-demand testing of a single zero-passage specimen banked in multiple replicate aliquots, setting SLiCE apart from organoid and precision-cut tumor explant models. Here, we describe results from our actively accruing clinical feasibility study (NCT05978557), where we successfully engrafted and tested 35 of 36 diverse brain tumor specimens on SLiCE, achieving our study’s primary endpoint ahead of schedule. SLiCE produced multi-parametric drug sensitivity scores (DSSs), each normalized to off-target toxicity, for all samples within a clinically actionable 28-day window. Across 530 experiments, we generated 142 DSSs from unique drug-tumor combinations, forming a reference library for future benchmarking. We then further analyzed a subset of IDH-WT glioblastoma tumor specimens in which SLiCE DSSs correlated with patient response to temozolomide (AUC = 0.875, p = 0.0175) and overall survival (R^2^ = 0.73). Additionally, this study validated surgically aspirated tumor tissue as a genomically, transcriptomically, and functionally similar tumor source compared to the standard, manually excised remnant tumor sample approved by clinical pathology. Collecting this often-discarded tumor source increased the mass of tumors accrued by nearly 5-fold and enabled collection from 11 additional patients, significantly increasing tumor tissue for downstream testing on SLiCE. These findings establish SLiCE as a scalable, clinically relevant platform for FPM in brain cancer, with potential to guide individualized treatment decisions and accelerate preclinical drug development.

## Introduction

Precision medicine aims to match therapies to the molecular features of individual tumors. However, in central nervous system (CNS) tumors, genomic profiling often fails to identify actionable targets or predict treatment response^1–5^. Functional precision medicine (FPM) offers a complementary approach by testing patient-derived tumor tissue *ex vivo*, enabling direct assessment of drug efficacy in a biologically relevant context^6^.

FPM has shown clinical utility across hematologic malignancies, solid tumors, and rare cancers^7,8^. Trials such as EXALT demonstrate that ex vivo drug sensitivity testing can guide treatment decisions, identify effective off-label therapies, and improve outcomes for individuals with blood cancers^8^. These leading platforms^9–12^ often grow patient tumor tissues *ex vivo* as organoids, spheroids, or precision-cut tumor explant cultures that each have their own limitations and varying abilities to (1) establish and test a broad range of high- and low-grade tumor types; (2) directly test passage-zero tumor tissue; (3) measure off-target toxicities of tested therapeutics; (4) complete drug sensitivity testing in a clinical actionable timeframe; and (5) cryopreserve tumor tissues for delayed, iterative, and/or on-demand testing^7,13,14^. These limitations are amplified in brain cancer, where tissue is scarce and the tumor microenvironment is complex.

Over the last decade, we have repurposed rat-derived organotypic brain slice cultures (OBSCs), long-used to study neurodegeneration and stroke by members of our team^15–32^, as living tissue substrates to engraft living human brain tumors and facilitate CNS tumor research^33–39^. This Screening Live Cancer Explants (SLiCE) technology (1) reproducibly engrafts viable, passage-zero patient brain tumor tissue, including low- and high-grade, pediatric and adult, primary and metastatic tumors; (2) recapitulates intrinsic tumor characteristics such as genomic fidelity, growth, invasion, and response to treatment; (3) incorporates tumor-associated immune cells (e.g., T cells and macrophages) and living OBSC-resident cells (e.g., astrocytes and microglia) to create a living TME; (4) generates multi-parametric drug response readouts as early as five days post-surgery, (5) and is amenable to engraftment of cryopreserved tumor tissues^33,40^. In July 2023, we opened a clinical feasibility study (NCT05978557)^41^ to assess the ability of SLiCE to engraft, treat, and analyze drug sensitivity profiles of diverse patient CNS tumor tissues. Here, we present the results of that study, which has met its primary objective ahead of schedule: SLiCE generated personalized Drug Sensitivity Scores (DSSs) within 28 days of resection in 35 of 36 patient tumor samples.

## Results

### Patient Characteristics and Study Design

**Figure 1:**
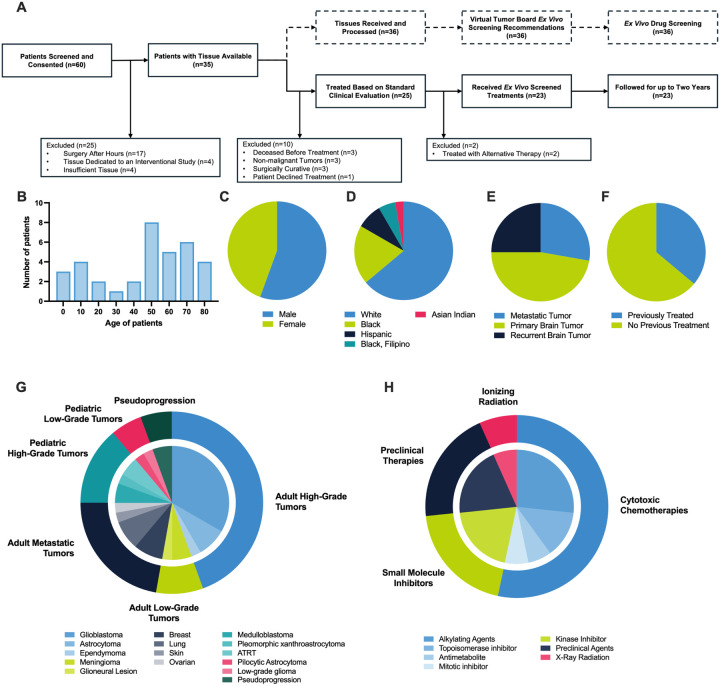
Patient Characteristics and Study Design (A) CONSORT diagram depicting patient screening and enrollment in a prospective, single-institution clinical feasibility study (NCT05978557). (B) Distribution of patient ages at the time of sample collection. (C) Sex distribution of patients contributing samples. (D) Racial demographics of patients contributing samples. (E) Tumor occurrence (primary vs. recurrent vs. metastatic) among patients. (F) Prior treatment status of patients at the time of sample collection. (G) Summary of tumor histologies represented in the study cohort. (H) Overview of therapeutic agents tested on organotypic brain slice cultures (OBSCs) within the study.

We conducted a prospective, single-institution feasibility study (NCT05978557)^41^ to evaluate the SLiCE assay using tumor tissue collected from patients undergoing standard-of-care neurosurgical tumor resection. Viable tissue was obtained from 35 of 60 consented patients (58%) (Fig. 1A). Barriers to acquisition included surgeries completed outside research hours (n = 17), tissue prioritization to other interventional trials (n = 4), and insufficient residual tissue for research (n = 4). One patient underwent a second resection, yielding matched samples from two time points.

Tumors were collected from patients aged 1 to 80 years (Fig. 1B). Among those with tissue received, 44% were female and 56% male (Fig. 1C). The cohort was demographically diverse, reflecting the catchment area of our Comprehensive Cancer Center: 62% White (n = 21), 21% Black or African American (n = 7), 9% Hispanic (n = 3), 6% Black or African American and Filipino (n = 2), and 3% Asian Indian (n = 1) (Fig. 1D). Tumor types included newly diagnosed primary brain tumors (44%), recurrent primary brain tumors (26%), and a mix of brain metastases (29%) (Fig. 1E). Based on clinical history, 38% of samples were obtained at tumor recurrence and were not treatment naive (Fig. 1F).

Within 2 days of resection, our virtual tumor board reviewed each patient’s clinical history, imaging findings, and preliminary pathology diagnosis to recommend a panel of drugs to be tested on the patient’s tumor using the SLiCE assay. All 36 samples received individualized drug recommendations from the tumor board and were tested using the SLiCE assay. All 36 samples underwent testing before the patient received post-surgical tumor-directed treatment; two of the samples were resected from the same patient during two different resection surgeries, resulting in the 36 samples representing 35 patients. Of the 35 patients, 25 received post-surgical treatment, while 10 received no additional treatment. All patients but one received drugs included in our screening panel, highlighting our panel’s clinical relevance (Fig. 1A). The cohort encompassed a broad range of CNS tumors, including adult and pediatric high- and low-grade tumors, brain metastases, and two pseudoprogression cases, demonstrating SLiCE’s adaptability across histologic and molecular subtypes (Fig. 1G). The therapeutic panel included 15 agents spanning cytotoxic chemotherapies, small-molecule inhibitors, preclinical compounds, and ionizing radiation (Fig. 1H).

### Primary and Secondary Endpoints: Patient Seeded SLiCE Assay and DSSs

**Figure 2:**
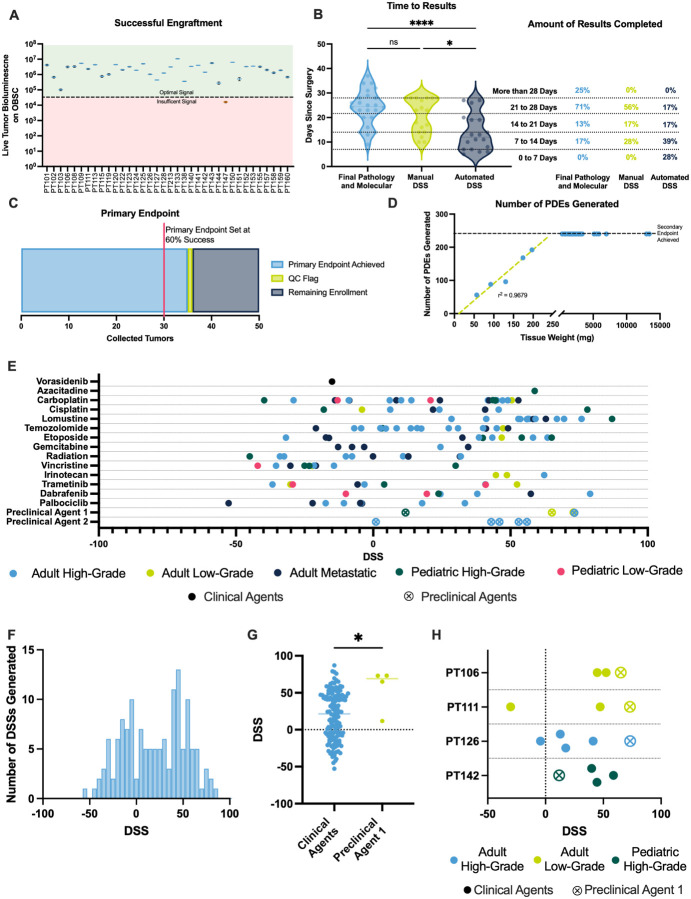
Primary Objective Outcomes from NCT05978557 (A) Bioluminescence signal intensity measured on day 4 from all tumor samples received. (B) Time to result for clinical pathology, molecular diagnostics, manual drug sensitivity score (DSS) calculations, and automated DSS outputs. Statistical analysis performed using ordinary one-way ANOVA with Tukey’s multiple comparisons test. *p < 0.0332, **p < 0.0021, ***p < 0.0002, ****p < 0.0001. (C) Number of tumors meeting the study’s primary endpoint. (D) Number of patient-derived explants (PDEs) generated per sample, normalized to initial tissue weight (mg). (E) Reference library of all high-quality DSSs generated in the study. (F) Distribution of DSSs across all samples. (G) Comparison of high-quality DSSs for clinical agents versus preclinical agent 1. Statistical analysis performed using unpaired t-test. *p < 0.0332, **p < 0.0021, ***p < 0.0002, ****p < 0.0001. (H) DSSs for each patient tested against preclinical agent 1.

The primary objective of this study was to evaluate the feasibility of engrafting diverse CNS tumor subtypes onto SLiCE and returning individualized drug sensitivity scores (DSSs) within a clinically relevant 28-day timeframe. Regardless of patient age, tumor grade, or tumor type, 35/36 (97%) of tumor specimens could be optimally maintained on SLiCE for downstream drug sensitivity testing (Fig. 2A). In the single sub-optimal sample, our quality control metrics worked as designed, independently flagging the sample and identifying an incubator malfunction unrelated to the SLiCE technology. Given the sub-optimal signal, the sample was not included in the downstream analysis.

For the 36 surgeries contributing tumor tissues to this study, time to receive standard clinical pathology and molecular testing results averaged 26 days after surgery (range: 14–37), with 25% of patients waiting more than 28 days for results (Fig. 2B). In contrast, our SLiCE-based DSS, which incorporates both on-target efficacy and off-target toxicity to measure “therapeutic windows” along several features of the dose-response curve, provided personalized data in an average of 22 days (range: 10–28), with DSSs for all patients calculated in less than 28 days. To decrease timelines and increase precision, we implemented automated analysis pipelines^42^, which produced DSSs in an average of 12 days (range: 8–27), with nearly 70% of automated results calculated within two weeks, and over 30% within one week; markedly faster than clinical testing, where over 75% exceeded three weeks and none were completed within one week (Fig. 2B). This acceleration was achieved by automating data analysis and minimizing manual effort.

Our primary endpoint was defined as calculating DSSs in ≤ 28 days for at least 60% of the first 50 collected tumor samples. Because we successfully calculated DSSs in 35 of our first 36 samples, we therefore met the primary endpoint ahead of schedule (Fig. 2C).

The secondary objective was to assess SLiCE’s scalability by evaluating whether the number of seeded microtumors linearly correlated with accrued tissue mass up to a limit of testing 240 microtumors per patient. A linear relationship was observed in cases where 250mg of tissue was accrued, showing that from patient to patient, a known tumor mass could be used to estimate the number of microtumors that could be generated (~ 1mg of patient tumor tissue per seeded microtumor; Fig. 2D). Interestingly, during this study, our laboratory and neurosurgical teams developed a method to collect much more tumor tissue into our lab than previously expected. In 86% of samples, we received much more tissue than was needed to generate the maximum 240 PDE threshold (quantified in Fig. 5, discussed later).

### Analysis of Drug Sensitivity Testing Results and Construction of a Reference Library

We next established a patient tumor drug sensitivity reference library based on SLiCE DSSs. Across 36 specimens, we completed 343 unique experiments. Replicate experiments testing the same therapeutic agent were aggregated, yielding 142 DSSs from unique single-agent drug-tumor combinations (Fig. 2E). Each tumor was tested against 1 to 8 agents, selected based on individualized recommendations from our virtual tumor board. DSSs ranged from − 52.7 to 87 (Fig. 2F), showing the breadth of patient tumor responses to different compounds. This dataset serves to show the feasibility of SLiCE and as a foundational reference for benchmarking novel therapies and correlating functional responses with clinical outcomes.

SLiCE-based DSSs can be used to evaluate the potential of experimental anticancer agents in the preclinical setting. To demonstrate this capability, we compared DSSs from a novel preclinical agent, Preclinical Agent 1, to all FDA-approved agents tested across the cohort. Preclinical Agent 1 showed significantly higher efficacy, with a mean DSS of 55.8 versus 17.5 for clinical agents (p = 0.0196; Fig. 2G), highlighting SLiCE’s ability to identify promising candidates that outperform current standards directly against zero-passage patient tissue.

SLiCE can also enable patient- and drug-specific sensitivity insights by comparing a set of therapeutics against a single patient’s tumor. Among four patient tumors tested with Preclinical Agent 1 on SLiCE, three exhibited their highest DSS with this agent, suggesting strong predicted sensitivity, while one tumor was non-responsive, with a DSS for that agent lower than all others (Fig. 2H). In this way, SLiCE can help identify relative responders and non-responders to specific therapies.

### Correlation of SLiCE DSS to Matched Patient Outcomes

**Figure 3:**
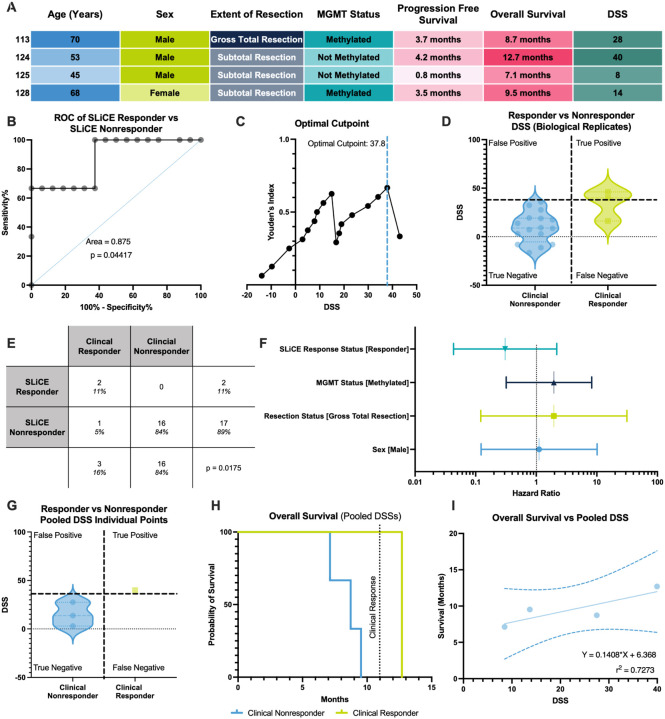
Clinical Utility of Functional Testing (A) Demographic characteristics of the temozolomide-treated glioblastoma cohort used for downstream analysis. (B) Receiver operating characteristic (ROC) curve comparing individual drug sensitivity scores (DSSs) between responders and nonresponders; area under the curve (AUC) = 0.875, *p* = 0.044. (C) Youden’s J index for individual DSSs in the glioblastoma cohort. (D) Distribution of individual DSSs stratified by clinical response status. (E) Contingency table comparing DSSs between responders and nonresponders; Fisher’s exact test, *p* = 0.0175. (F) Hazard ratios for MGMT methylation status, extent of resection, sex, and DSS, calculated using log-rank tests. (G) Distribution of pooled high-quality DSSs stratified by response status. (H) Kaplan–Meier analysis of overall survival in responders versus nonresponders. (I) Correlation between pooled high-quality DSSs and overall survival.

The study recruited a heterogeneous cohort of primary and metastatic CNS tumors, with patients receiving a wide range of single-agent and combination therapies. Though unpowered, we evaluated DSS as a predictive biomarker for TMZ response by analyzing the four glioblastoma (GBM) patients who met the following criteria: (1) received temozolomide (TMZ) post-surgery; (2) did not receive any targeted inhibitors; (3) had TMZ DSSs; and (4) had ≥ 11 months of follow-up. Despite the small sample size, this cohort provided meaningful insights into SLiCE’s clinical utility. Demographic and clinical variables, including age, sex, extent of resection, and MGMT methylation, did not distinguish responders from nonresponders (Fig. 3A), underscoring the need for improved biomarkers. Patients surviving ≥ 11 months were classified as responders, with others as nonresponders, as reported before^43^. To increase statistical power for DSS cut-point calculations, we conducted multiple rounds of TMZ testing on each patient’s tumor, using multiple cryopreserved aliquots of each specimen as biological replicates. Receiver Operating Characteristics (ROC) curve analysis yielded an area under the curve (AUC) of 0.875 (p = 0.044), indicating strong predictive performance in distinguishing responders from nonresponders (strong performance designated by AUC > 0.8^44^ Fig. 3B). Youden’s J index^44,45^, a summary statistic used to evaluate performance of a diagnostic test, identified a DSS optimal threshold of 37.8 for binary classification (Fig. 3C), which cleanly separated responders from nonresponders, with no false positives and one false negative by balancing sensitivity and specificity (Fig. 3D). Concordance between SLiCE-predicted and actual outcomes was 95%, confirmed by Fisher’s exact test^46^ (p = 0.0175; Fig. 3E).

To contextualize these findings, we compared hazard ratios derived from SLiCE response status to other clinical variables using log-rank tests. Despite wide confidence intervals, SLiCE response status, as quantified by a DSS, was associated with a hazard ratio of 0.31, suggesting a potential protective effect (Fig. 3F). MGMT methylation, resection status, and sex showed weaker associations with survival.

We also evaluated pooled DSS values averaged across replicates, representing the clinical reporting format. Using this approach, all patients were correctly classified as responders or nonresponders (Fig. 3G). Kaplan–Meier analysis supported SLiCE’s clinical relevance: the single SLiCE DSS responder, with a score just above the cut-point, showed extended survival just beyond 11 months, while all nonresponders experienced earlier mortality (Fig. 3H). Although not statistically significant due to sample size, this trend in patient survival aligned with SLiCE predictions. A strong correlation between DSS and overall survival in months (two continuous variables, R^2^ = 0.73) suggests DSS may predict both categorical response and survival duration (Fig. 3I). Importantly, the single responder was barely over the DSS cut-point and survived just beyond the clinical threshold.

### Iterative Testing and Combination Therapy on a Single Patient’s Tumor

To further assess patient-level insights, we analyzed GBM patient PT113 as a case study to perform iterative single-agent and combination therapy within the standard 28-day window between tumor resection surgery and treatment initiation. Using our standard 4-day SLiCE assay, we generated functional drug sensitivity readouts from eight single-agent therapies, and then rationally combined multiple therapies in a second round of testing based on the initial single-agent testing data.

**Figure 4:**
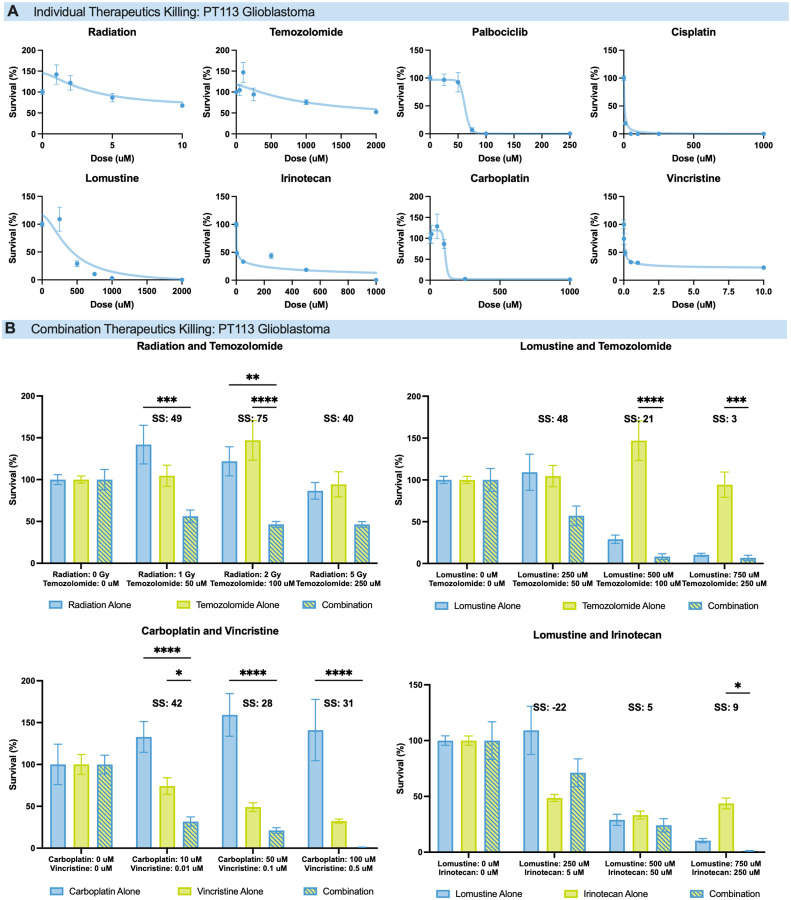
Patient-Specific Utility of Functional Testing (A) Drug sensitivity profile for patient PT113, tested against radiation, temozolomide, palbociclib, cisplatin, lomustine, irinotecan, carboplatin, and vincristine (n = 4 wells per dose per agent). (B) Combination therapy testing for PT113, an adult glioblastoma case. Synergy scores (ZipSynergy, SS) are reported above each drug combination. Statistical analysis performed using two-way ANOVA. *p < 0.05, **p < 0.01, ***p < 0.001, ****p < 0.0001.

In the initial round of testing, PT113 was screened against external radiation, TMZ, palbociclib, cisplatin, lomustine, irinotecan, carboplatin, and vincristine (Fig. 4A). At doses with incomplete monotherapy killing, single agents were combined into clinically relevant regimens for a second round of testing using a second cryopreserved aliquot of PT113 tumor tissue: lomustine/TMZ, TMZ/radiation, lomustine/irinotecan, and carboplatin/vincristine (Fig. 4B).

Synergy was quantified using SynergyFinder^47,48^ and the Highest Single Agent (HSA) model^49^: SS < − 10 indicates antagonism, − 10 < SS < 10 indicates an additive effect, and SS > 10 indicates synergy. TMZ/radiation and carboplatin/vincristine showed strong synergy across all doses. Lomustine/TMZ showed high synergy at low doses, tapering to additive effects at higher concentrations. Lomustine/irinotecan exhibited antagonism at low doses and additive effects at higher ones.

#### Surgically Aspirated Tumor Tissue is a Viable and Equivalent Tumor Source Compared to Manually Excised Tissue

Obtaining viable CNS tumor tissue for functional drug screening is challenging, especially in the preclinical setting. To overcome this persistent challenge, we evaluated surgically aspirated tumor tissue as a viable and scalable source for *ex vivo* drug screening. Unlike tumor specimens removed in a piecemeal fashion and sent to pathology (and eventually to downstream research), “aspirate” is commonly removed via ultrasonic tissue disruption and aspiration during tumor debulking and often discarded, due to concerns about viability and molecular fidelity^50–52^. Because aspirate is not desired by clinical pathology, this tissue source can be brought directly to our laboratory for testing. In this feasibility study, we implemented a dual-source strategy, incorporating both standard excisional tissue (sPT) and aspirate into the SLiCE workflow.

**Figure 5:**
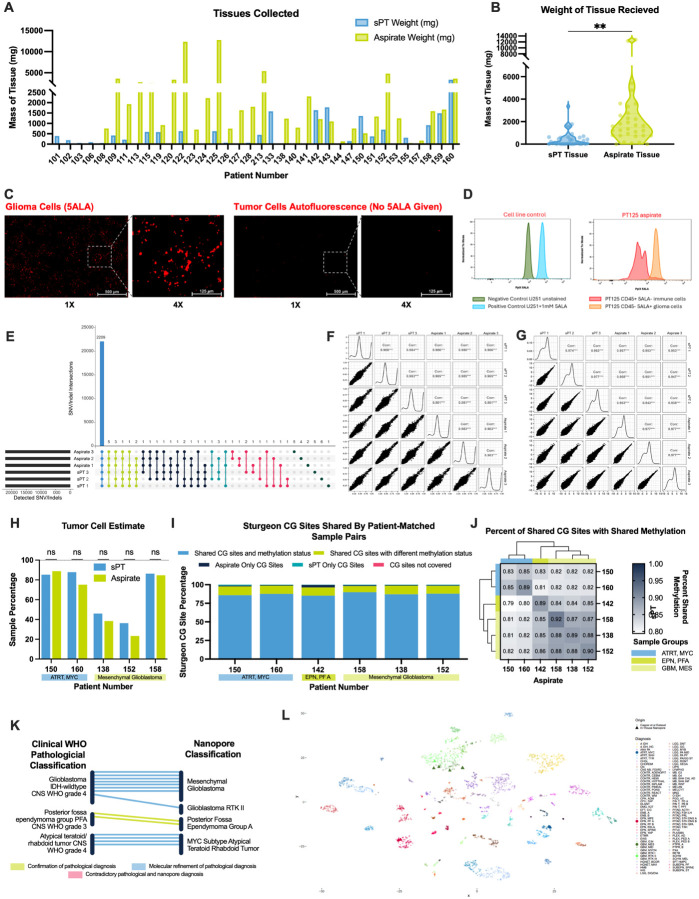
Biologic and Genetic Similarity of Aspirate and sPT Samples (A) Amount of sPT or aspirate tissue received per patient (mg). (B) Average tissue yields across the collection period. (C) Confirmation of glioblastoma tumor cells in a tissue removed via aspiration following 5-ALA administration by fluorescence imaging. (D) Confirmation of glioblastoma tumor cells in a tissue removed via aspiration following 5-ALA administration by flow cytometry. (E) Variant concordance between replicates of two distinct tissue collection methods – standard preclinical and aspirate. UpSet plot showing intersection of SNVs (VAF ≥5%) and Indels (VAF ≥10%) detected using whole exome sequencing at depth of coverage ≥100X in *all* six samples. As shown in the figure >99.99% of detected SNVs and Indels are concordant across all samples. (F) Pairwise scatter plots and Pearson’s correlation (r) of the underlying VAFs of detected SNVs and Indels between replicates of two distinct tissue collection methods. (G) Pairwise scatter plots and Pearson’s correlation (r) of RPKM values of gene expression profiles obtained from whole transcriptome sequencing for replicates of two distinct tissue collection methods – standard preclinical and aspirate. (H) Estimated tumor purity in matched samples assessed by nanopore sequencing. (I) Shared Sturgeon CG sites in six matched samples assessed by nanopore sequencing. (J) Heatmap of the percent sharded CG sites with shared methylation status of 12 samples. (K) Classification of 14 samples by both the pathological WHO classification and nanopore classification. (K) t-SNE of 14 2212 collected patient samples onto historical background dataset for confirmation of classification.

Among 36 samples, aspirate yielded significantly more tissue than sPT (mean: 2,375 mg vs. 502 mg; paired t-test, p-value: 0.0014), with 29 samples providing greater volume from aspirate than from sPT (Fig. 5A–B). Without aspirate collection, 53% of samples would have fallen below the 240 mg threshold required to test a full 10-drug panel on SLiCE; with aspirate, only two samples remained below this threshold, both of which were aspirate-only collections. Notably, 11 samples were obtained exclusively from aspirate: sPT from these consenting patients was not available because (1) surgeries were completed after normal working hours, and sPT was held in pathology overnight; (2), sPT was allocated to other studies, or (3) there was insufficient residual sPT for research.

We first confirmed tumor cell presence in aspirate from a high-grade glioma (HGG) using protoporphyrin IX (PpIX) fluorescence. Surgeons sometimes administer 5-aminolevulinic acid (5-ALA), a heme precursor, to enable fluorescence-guided resection. HGG cells lack ferrochelatase, leading to PpIX accumulation and tumor-specific fluorescence^53–55^. A patient received 5-ALA prior to surgery, and microscopy of the resected aspirate revealed abundant PpIX signal, confirming tumor cell presence (Fig. 5C). In a separate experiment, we first validated a flow cytometry-based approach to measure 5-ALA-driven fluorescence in the human GBM cell line U251. We then used passage-zero patient GBM tissue (specimen PT125) to quantify the presence of both 5ALA^+^/CD45^−^ tumor cells and 5ALA^−^/CD45^+^ immune cells in PT125 (Fig. 5D). These methods confirm aspirate contains viable tumor and non-tumor cells suitable for downstream analysis.

Our standard SLiCE workflow uses a cryopreservation protocol to store representative aliquots of each collected tumor tissue. To measure whether each cryopreserved aliquot of tumor was molecularly similar, we performed bulk whole exome and whole transcriptome sequencing (WES and WTS) on six aliquots obtained from a single patient: three aliquots of sPT and three aliquots of aspirate. Variant calling from WES revealed > 99.99% concordance in SNVs (Variant Allele Frequency, VAF, ≥ 5%) and Indels (VAF ≥ 10%) across all six samples (Fig. 5E), with Pearson’s correlation coefficients for VAFs ranging from 0.983 to 0.989 (Fig. 5F). WTS showed similarly high reproducibility in gene expression profiles, with Reads per Kilobase of transcript per Million mapped reads (RPKM)-based pairwise correlations ranging from 0.938 to 0.977 (Fig. 5G).

We further assessed biological similarity between sPT and aspirate using nanopore-based methylation profiling. Downsampling was used to minimize coverage-dependent differences. Because patient tumor samples contain both tumor cells and tumor associated cells, we first measured differences in the proportion of tumor cells present within matched sPT and aspirate samples. Tumor cell estimates, derived from reads per megabase across tumor-specific chromosomes, downsampled to the lowest genomic coverage in the experiment, revealed no significant differences between matched aspirate and sPT samples (Fig. 5H). We did observe patient-to-patient variability in tumor cell estimates, reflecting differences in patient-specific tumor microenvironment content. Using a 450k CpG site set^56^, we found that patient-matched samples of sPT and aspirate share an average of 97.9% (range 96.3–98.6%) of confidently covered CG sites, regardless of methylation status, and that 87.2% (range 85.3–89.8%) of shared sites also shared methylation status, indicating high concordance (Fig. 5I).

Aspirate and sPT samples collected from the same patient exhibited strong methylation correlations, while inter-patient samples from the same methylation class (e.g., mesenchymal GBM) showed moderate inter-patient correlation (Fig. 5J). Notably, one posterior fossa group A ependymoma sample correlated more closely with mesenchymal GBM than with MYC-subtype ATRT, a finding suggestive of complex epigenetic relationships.

To further explore concordance between nanopore classification and clinical diagnosis of each tumor specimen, we measured each patient’s tumor separately and expanded the cohort to include two additional aspirate samples. These analyses utilized full genome coverage with no downsampling, reflecting existing clinical protocols. In 12 of 14 specimens, nanopore classification of the tumor tissues matched the clinical diagnosis and provided further molecular refinement. In the other two specimens, nanopore classification exactly matched the clinical diagnosis. There were no instances of discordant diagnoses between pathological and nanopore-based classification (Fig. 5K).

Finally, we compared the classification determined by our nanopore sequencing to a historic cohort. We used a validated probe set^56^ and re-embedded our samples into the Capper et al t-SNE space^57^ for high-dimensional visualization to meaningfully resolve the complex quantitative relationships. In-house nanopore profiles (triangles) projected onto the reference t-SNE clustered within expected diagnostic regions, with ATRT and EPN PF A samples aligning to their respective groups and GBMs mapping to distinct molecular subtypes (Fig. 5L). No samples appeared as outliers or with classifications between distant classes.

Together, the results presented in Fig. 5 demonstrate that (1) sPT and aspirate are both representative of the same specimen; and (2) cryopreserved aliquots of tumor tissue stored for downstream functional testing on SLiCE are similar to each other, providing confidence that multiple rounds of testing can be completed multiple aliquots of a single patient’s tumor.

### Validation of Aspirate as a Tissue Source for Functional Drug Screening on SLiCE

**Figure 6:**
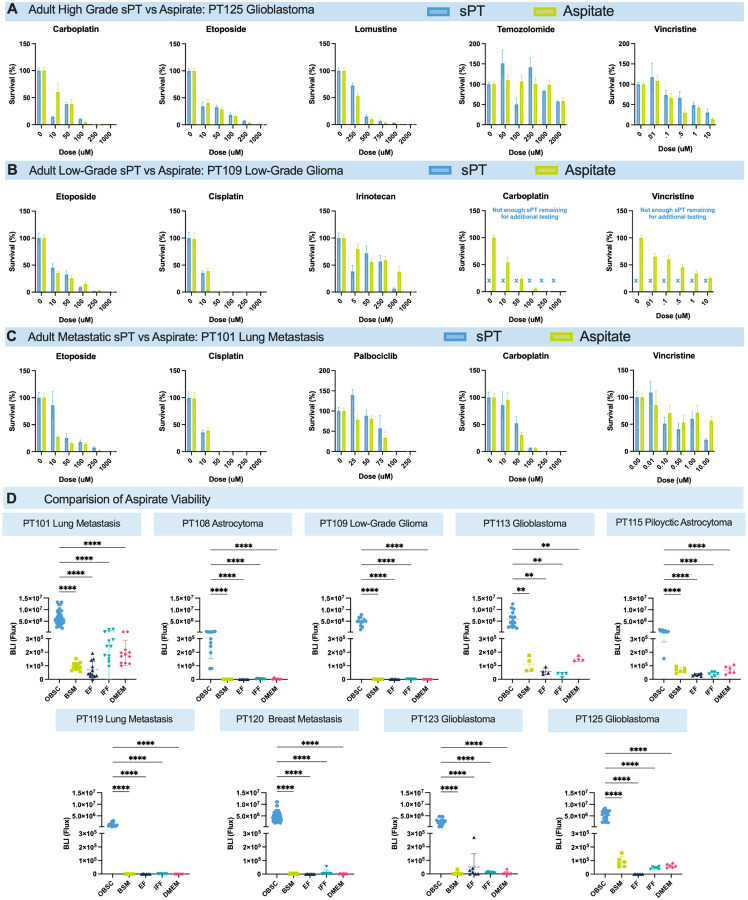
Exploratory Objectives: Functional Response of Aspirate Samples (A) Drug response comparison between patient-matched aspirate and sPT from PT125, an adult highgrade tumor. Agents tested included carboplatin, etoposide, lomustine, temozolomide, and vincristine. No statistically significant differences were observed (two-way ANOVA; n = 4 per dose, per drug, per tissue type; p > 0.05). (B) Drug response comparison between patient-matched aspirate and sPT from PT109, an adult low-grade tumor. Agents tested included etoposide, cisplatin, and irinotecan. Additional testing of vincristine was performed on aspirate due to increased tissue volume. A significant difference was observed at 10 μM cisplatin (two-way ANOVA; p = 0.0157); all other comparisons were not statistically significant (n = 4 per dose, per drug, per tissue type; p > 0.05). (C) Drug response comparison between aspirate and sPT from PT101, an adult metastatic tumor. Agents tested included etoposide, cisplatin, palbociclib, carboplatin, and vincristine. No statistically significant differences were observed (two-way ANOVA; n = 4 per dose, per drug, per tissue type; p > 0.05). (D) Viability of aspirate samples from 9 distinct tumor types after four days in culture. Samples were cultured under five conditions: (1) engraftment on OBSCs with specialized BSM beneath transwell; (2) in vitro culture in BSM; (3) in EF media; (4) in IFF media; and (5) in DMEM + 10% FBS. Aspirate survival on OBSCs was significantly higher than in all other media conditions (one-way ANOVA; n = 6; ****p < 0.0001; **p < 0.002).

Encouraged that aspirate is comparable genomically and epigenetically to sPT, we sought to validate aspirate as a functional surrogate for sPT. We first evaluated drug sensitivity concordance between aspirate and sPT collected from the same patient during the same surgery. Fifteen drug–tumor combinations were tested across three tumor types: a GBM (PT125, Fig. 6A), a low-grade glioma (PT109, Fig. 6B), and a lung tumor metastatic to the brain (PT101, Fig. 6C). Drug sensitivity trends among all sPT vs aspirate comparisons were similar; the average percent difference in tumor kill across all doses tested was 2.9%, with the highest differences occurring near the IC50 of some therapeutics. In PT109, we received much less sPT than we did aspirate, limiting the number of experiments we could complete with sPT alone. While the carboplatin and vincristine vs PT109 data we show here does not compare sPT to aspirate, it does highlight how the larger amounts of aspirate tissue we receive enable more drug sensitivity testing than sPT.

Next, we compared the viability of aspirate on SLiCE vs its viability after incubation *in vitro*. Equivalent amounts of aspirate tumor tissue collected from 9 different patients were cultured on SLiCE and in four media formulations: (1) the media used to culture tumor-bearing OBSCs (BSM); (2) serum-free neurobasal media with EGF and FGF (EF); (3) a neurobasal-based media recipe used by the Ian’s Friends Foundation (IFF); and (4) DMEM with 10% FBS. EF and IFF media recipes are optimized for brain cancer cell lines and organoids^58–61^. Across all conditions and tumors tested, SLiCE supported markedly higher tissue viability, even when tumor maintenance *in vitro* completely failed, highlighting its capacity to preserve heterogeneous tumor tissue, including fragile and lower-grade samples (Fig. 6D). Overall, these data show that aspirate maintains viability on SLiCE, enables reproducible profiling, and offers a scalable tissue source for individualized assessment.

### Assay Reproducibility and Quality Control

To ensure reliability and scalability, we implemented a comprehensive quality control (QC) framework focused on tissue processing, assay reproducibility, and platform robustness. These evaluations validated the consistency of drug sensitivity results across handling conditions and time points, establishing standards for clinical translation.

**Figure 7:**
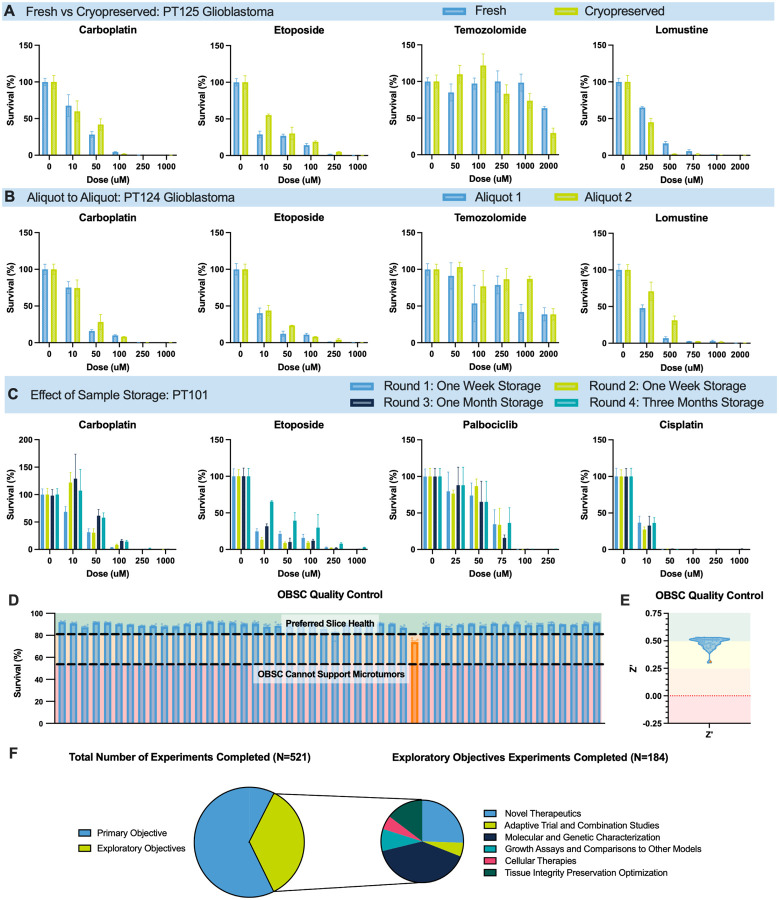
Quality Control Metrics and Reproducibility (A) Comparison of drug responses from fresh versus cryopreserved/thawed aspirate from PT125, engrafted on OBSCs. Agents tested included carboplatin, etoposide, lomustine, and temozolomide (n = 4 per dose, per drug, per condition). No statistically significant differences were observed (two-way ANOVA; p > 0.05). (B) Reproducibility of drug responses across two separate aliquots of PT124, engrafted on OBSCs. Agents tested included carboplatin, etoposide, lomustine, and temozolomide (n = 4 per dose, per drug, per aliquot). No statistically significant differences were observed (two-way ANOVA; p > 0.05). (C) Stability of cryopreserved PT101 aspirate over three storage durations in liquid nitrogen: one week, one month, and three months. Samples were tested against carboplatin, cisplatin, etoposide, and palbociclib (n ≥ 4 per dose, per drug, per time point). No statistically significant differences were observed (two-way ANOVA; p > 0.05). (D) OBSC viability over time, assessed by propidium iodide staining. (E) Z′-factor values of OBSCs used throughout the study, indicating assay robustness. (F) Total number of experiments conducted addressing both primary and exploratory objectives. Exploratory studies included testing of novel therapeutics, adaptive trial, and combination strategies, molecular and genetic characterization, growth assays, cellular therapies, and tissue integrity preservation.

We first assessed whether cryopreservation affected aspirate function. Matched fresh and cryopreserved aliquots from a GBM patient (PT125) were tested against four chemotherapeutics. No significant differences were observed, displaying how functional integrity of aspirate is preserved after cryopreservation (Fig. 7A). This cryopreservation protocol enables flexible scheduling, batch processing, and long-term biobanking.

To evaluate functional reproducibility among aliquots, two cryopreserved aliquots from a patient GBM (PT124) were processed and tested in parallel. Drug responses were consistent across agents, indicating that each aliquot is representative and suitable for longitudinal or multi-condition experiments (Fig. 7B).

We next examined the stability of cryopreserved tissue over a three-month period. Using aspirate from a patient with metastatic lung cancer (PT101), we conducted 16 experiments across four time points over three months. Drug responses remained stable, confirming viability for delayed testing and retrospective analysis (Fig. 7C).

Maintaining OBSC health is essential for SLiCE reliability. We have therefore implemented a QC metric using propidium iodide staining, which measures viability of the cells within each OBSC. For every batch of ~ 150 OBSCs we generate, we dedicate six randomly selected OBSCs for QC testing. We have established that OBSCs with overall viability lower than 54% are no longer able to support patient tumor engraftment and have set a QC threshold far above that mark, desiring OBSCs with 80% viability for downstream testing. All but one batch of OBSCs generated during this study hit this mark, with the one exception helping us identify an incubator malfunction (Fig. 7D, orange bar). This issue was flagged in real time and corresponded with the suboptimal signal in PT147 (Fig. 1D), confirming the cause was technical, not biological.

To complement biological QC, we used the Z’ factor to assess assay robustness. Across OBSC runs, 46% achieved Z’ >0.5 (exceptional), and 54% fell within the acceptable range of 0.25–0.5 (Fig. 7E). The flagged OBSC batch had one of the lowest Z’ scores, reinforcing the utility of this metric for identifying compromised assays (Fig. 1D, Fig. 7D–E).

The increase in collected patient tumor tissue afforded by aspirate collection, combined with its confirmed genetic and functional similarities to sPT, drastically increased the number and types of experiments that we were able to complete. We completed 521 experiments during the clinical feasibility study, including 184 addressing exploratory objectives (Fig. 7F). These included testing novel therapeutics such as preclinical agents, cell therapies, and combination regimens, aligning SLiCE results to molecular profiles, conducting tumor growth assays, and optimizing the SLiCE assay to maximize tissue integrity. Exploratory studies were completed on 76% of samples, underscoring SLiCE’s capacity for comprehensive functional interrogation.

## Discussion and Conclusions

Where genomics-based precision medicine has fallen short, FPM offers significant promise for truly personalized treatment strategies. This study demonstrates the feasibility, scalability, and timely relevance of the SLiCE platform and assay. This rapid *ex vivo* drug screening assay can be integrated into real-time pre-clinical and clinical workflows and offers a framework for individualized therapy selection in brain tumor patients, who often suffer from limited treatment options and poor outcomes. SLiCE’s use of passage-zero patient tumor tissue, alongside its fast turnaround times, cryopreservation-amenable workflows, normalized DSS readouts, and maintenance of features in the tumor microenvironment overcome key translational barriers seen in other models, enabling SLiCE to help guide both pre-clinical and clinical decision-making.

In our prospective study, we have thus far accrued tumor tissues from 58% of consented patients and returned DSSs for all collected samples, with one sample failing QC parameters due to an incubator failure. The primary endpoint – returning DSSs within 28 days after surgery for ≥ 60% of the first 50 collected tumor specimens – was met upon accrual of the first 31 patients. Automated DSS pipelines reduced turnaround time and manual effort, enhancing SLiCE’s suitability for routine clinical use^42^. Compared to standard pathology and molecular testing ordered in the clinic, SLiCE cut time to results by over 50%, enabling earlier, informed treatment decisions during critical care windows. The cohort was demographically diverse and representative of North Carolina’s population, supporting SLiCE’s broad applicability. SLiCE consistently enabled engraftment and DSS generation across diverse CNS tumor subtypes.

We also met our secondary objective, which was to test whether we could linearly scale the amount of tumor testing conducted as a function of the amount of tumor mass collected, up to 240 microtumors per patient. Before adding aspirate to our workflow, we did show this linear relationship (Fig. 1D). Incorporating aspirate as an additional tissue source provided so much more tissue for testing that we surprisingly hit that 240 microtumor threshold in 86% of cases and had leftover aliquots of tumor tissue remaining. Aspirate therefore addresses a major barrier in functional brain cancer models – limited tissue availability – and expands patient eligibility for ex vivo testing.

Although SLiCE results did not guide treatment decisions in this study, 96% of treated patients received agents included in our screening panel, reflecting strong alignment with clinical practice. This feasibility study was not positioned nor powered to measure DSS as a predictive biomarker for clinical response to the same drug, but we were able to estimate the correlation between TMZ DSS and clinical response in a small cohort of patients. In this limited cohort, SLiCE-derived DSSs for TMZ strongly correlated with clinical outcomes. Demographic and clinical variables, including age, sex, extent of resection, and MGMT methylation, did not reliably distinguish responders, highlighting limitations of current biomarkers. ROC analysis of the sensitivity and specificity of SLiCE DSSs yielded an AUC of 0.875, and a DSS threshold of 37.8, calculated by Youden’s index, that cleanly separated responders from nonresponders. These predictions were supported by contingency analysis (p = 0.0175), hazard ratio trends, and Kaplan–Meier curves. A continuous relationship between DSS and overall survival (R^2^ = 0.73) suggests SLiCE may predict not only categorical response but also survival duration – an insight with significant prognostic value. Validation in larger cohorts could enable more precise prognostication and treatment planning. These data demonstrate the feasibility and highlight SLiCE’s potential to guide personalized treatment and avoid ineffective therapies. By incorporating functional response data into clinical decision-making, SLiCE supports a more nuanced precision medicine model, one that considers both population-level trends and individual variability.

Building on individualized tumor board input, SLiCE enabled the development of a scalable, clinically relevant DSS reference library. As the library grows, it will support cell–type–specific analyses and iterative testing strategies, facilitating rational design of personalized single-agent and combination therapies. The library also serves as a benchmark for evaluating novel agents in a human-derived, clinically relevant context. Our analysis of DSSs from a preclinical compound against other drugs in the cohort, and also the relative efficacy of that drug against specific patient tumors, demonstrates SLiCE’s ability to identify promising therapeutic candidates, and which agents may be most – and least – effective against specific patient tumors. This capability can inform biomarker-driven trial design and enhance therapeutic precision.

SLiCE’s clinical utility was further demonstrated through iterative testing of patient PT113. Rapid turnaround enabled timely identification of effective monotherapies and synergistic combinations, supporting adaptive treatment planning. This case illustrates SLiCE’s ability to generate actionable insights within a clinically relevant timeframe and its potential to support dynamic, patient-specific drug screening.

Incorporating aspirate tissue, typically discarded during surgery, significantly increased tissue yield, enabling broader patient participation and deeper biological interrogation. Aspirate provided significantly greater masses of tumor tissue than sPT and showed equivalent viability and drug response, validating its use as a primary input. Furthermore, it showed genetic similarity on both a DNA and RNA level between representative aliquots and different extraction methods. Beyond practical advantages, aspirate enabled deeper insights into tumor composition.

Robust quality control measures ensured assay fidelity. Cryopreserved aspirate maintained functional integrity across time points, and aliquot-to-aliquot reproducibility confirmed sample consistency. Real-time QC metrics, including OBSC viability thresholds and Z’ factor analysis, enabled early detection of assay failures and reinforced reliability of results.

Recent regulatory shifts further support SLiCE’s relevance as a “New Approach Methodology”^62,63^. The FDA’s 2022 and 2025 decisions to prioritize human-based testing over animal models reflect growing recognition of limitations in traditional preclinical systems. SLiCE aligns with this shift by rapidly and directly measuring drug response in passage-zero patient tumor tissue. These findings position SLiCE as a transformative platform for precision medicine. Its rapid turnaround, scalability, and predictive accuracy support integration into clinical workflows, enabling more personalized and adaptive treatment strategies. As regulatory frameworks increasingly favor human-relevant testing in the preclinical setting, SLiCE is well-positioned to accelerate therapeutic development, guide individualized care, and redefine functional precision medicine for CNS tumors.

## Methods

### Study Design

This prospective, single-institution feasibility study (ClinicalTrials.gov: NCT05978557)^41^ evaluated the SLiCE assay, a rapid ex vivo drug screening platform using passage-zero brain tumor tissue seeded onto organotypic brain slice cultures (OBSCs). The study assessed assay feasibility, scalability, and turnaround time for individualized drug sensitivity scores (DSSs).

### Patient Enrollment

All brain tumor specimens were collected at University of North Carolina Hospitals through two UNC IRB biospecimen collection protocols. Patients undergoing neurosurgical resection for CNS tumors were eligible. Inclusion required viable tumor tissue and research consent. Exclusion criteria included tissue exhaustion for diagnostic or interventional use or surgeries outside research hours. No restrictions were placed on age, histology, or treatment history.

### Ethical Approval

Appropriate written informed consent by parent/guardian and assent as applicable was obtained under protocols where ethical approval was given by the Institutional Review Board (IRB) in the Office of Human Research Ethics at the University of North Carolina at Chapel Hill. Most patients consented to non-interventional IRB Protocol #23–0834 (LCCC2212; ClinicalTrials.gov NCT05978557), which began on July 27, 2023; estimated end date March 1, 2028. One patient (Fig. 5C) was consented under IRB Protocol #20–1878 approved by the University of North Carolina’s Institutional Review Board, which began on August 7, 2020; end/renewal date October 9, 2026. The laboratory study team had no access to identifying patient information; de-identified or coded diagnostic data describing tumor type and mutational status was provided by a Study Coordinator / Honest Broker. Written informed consent was obtained from adult participants; pediatric enrollment required parental consent and age-appropriate assent.

### Tissue Collection and Processing

Tumor tissue was collected intraoperatively and de-identified via an honest broker. Standard pathology tissue (sPT) was obtained from excisional material not required for diagnosis. Aspirate tissue was collected using suction-based tools. Both tissue types were washed in PBS, minced, and cryopreserved in CryoStor10 (1 mL per 150 mg)^33^. Aspirate samples underwent red blood cell lysis (1× RBC lysis buffer, 10 min, room temperature, repeated once). Samples were thawed in a 37°C water bath prior to use. A minimum of 50 mg was required for assay completion.

### SLiCE Assay

OBSCs were prepared from postnatal day 8 Sprague Dawley rat pups (Charles River Labs, RRID:MGI:5651135) under UNC IACUC protocols 22–171 and 25–142. Brain slices were cultured on inserts in 6-well plates with 1 mL of brain slice media at 37°C, 5% CO_2_, and 95% humidity^33^.

Patient tissue was transduced with a lentiviral vector expressing mCherry and firefly luciferase (fLuc) at 1×10^6^ viral particles per 150 mg. Transduced tissue (1 mg) was seeded onto each brain hemisphere. Drug exposure began on day 1 post-seeding. Therapeutics included monotherapies and combinations selected by oncology physicians. Drug concentrations were predefined and constrained by off-target toxicity assessed via propidium iodide staining. Viability was measured on day 4 using luciferin and an AMI optical imager (Spectral Instruments).

DSSs were calculated using manual and automated pipelines. The automated pipeline is established and explained in detail^42^. OBSC viability was monitored using propidium iodide staining; thresholds were ≥80% (preferred) and ≥55% (minimum).

### Drug Selection Workflow

A de-identified list of available therapeutics was provided to oncology physicians by the honest broker. Clinicians independently selected agents based on clinical relevance, histology, and treatment history. Drug recommendations were submitted via REDCap and blinded to SLiCE results.

The standardized drug panel included 13 agents: carboplatin, cisplatin, lomustine, temozolomide, etoposide, gemcitabine, vincristine, irinotecan, trametinib, dabrafenib, palbociclib, ionizing radiation, and two preclinical compounds. Additional agents could be suggested. Drug selections were compiled and transferred to the research team in a de-identified format.

### 5 ALA imaging

Fluorescent images of 5-ALA were obtained using a Thunder 3D Imager. Excitation wavelength: 390 nm Emission wavelength: 700 nm

### 5ALA treatment of control cell lines in vitro

A 500mM stock solution of 5-Aminolevulinic acid hydrochloride (Sigma-Aldrich A3785) was prepared in PBS. U251 glioma cell line was seeded on cell culture plates two days before 5ALA treatment. Cells were treated overnight with 1 mM 5ALA in cell culture media. The next morning, cells were lifted and prepared for flow cytometry analysis as a positive control.

### Flow Cytometry Tumor dissociation and debris removal

Patient tumor surgical aspirate or solid tissue was thawed by gentle agitation in a 37°C water bath. Tumor tissue was weighed and then digested with the Human Tumor Dissociation Kit (Miltenyi Biotec 130-095-929) according to the manufacturer’s instructions. The dissociated tissue was passed through a 100uM filter to remove any undigested tissue. Next, debris and myelin were removed by a Percoll gradient as previously described^64^. Briefly, the cells were resuspended in 30% percoll and a layer of 70% Percoll was underlaid using a Pasteur pipette. Samples were centrifuged for 20 minutes at 340xg with minimum acceleration and no brake to obtain the density gradient. After centrifugation, the debris layer was removed from the top, and the cells were extracted from the interface layer and placed in post-percoll solution (5% FBS with 10 uM HEPES in HBSS with Calcium and Magnesium). After centrifugation, cells were resuspended in PBS for antibody staining.

### Flow Cytometry Antibody staining

All antibodies were titrated in control cell lines and in PBMCs to select appropriate concentrations. After obtaining a single cell suspension from patient tumors, cells were stained with Zombie Aqua fixable viability dye (Biolegend 423102) in PBS for 15 minutes. Cells were then fixed using the FlowX FoxP3/Transcription factor fixation and permeabilization kit (R&D Systems FC012–100) according to manufacturer instructions. Human Trustain FCX (Biolegend 422301) was added for 5–10 minutes before surface staining. Cells were stained for 30 minutes with anti-CD45 PE-Cy7 (BD 560915) in PBS with TruStain monocyte blocker (Biolegend 426101). Cells were then washed and permeabilized using the FlowX FoxP3/Tramscription factor fixation and permeabilization kit (R&D Systems FC012–100) according to manufacturer instructions. After permeabilization, intracellular staining was performed. Cells were incubated overnight with Helix NP NIR DNA dye (Biolegend 425301) in 1:1 solution of permeabilization buffer and PBS. After overnight staining, cells were washed and resuspended in PBS for analysis. Appropriate single-color reference controls and full-stained positive controls using human PBMCs and U251 cell line were prepared. Samples were analyzed on the Cytek Aurora at the UNC flow cytometry core. Data were analyzed in Spectroflo. Peak emission of PpIX was in the V11 channel.

### Sample preparation and extraction for Whole Exome and Whole Transcriptome Sequencing

Three replicates from each removal method, sPT or aspirate tissue, were processed starting from aliquots stored at −80°C that had previously been minced and filtered with a 100 μm cell strainer before freezing. Nucleic acid extraction was carried out following the Qiagen AllPrep DNA/RNA Mini Handbook (Qiagen Cat#80204), using a TissueRuptor (120 V, 60 Hz, Qiagen, Cat# 9001271) for homogenization, and following the Protocol: Simultaneous Purification of Genomic DNA and Total RNA from Animal Tissues. Aliquots were removed from −80°C in batches and immediately layered with RLT Plus reagent that included β-mercaptoethanol (Sigma-Aldrich Cat#M3148) according to the manufacturer’s instructions. Tissue aliquots in buffer were inverted every few minutes to mix, and kept on ice until thawed. Inputs for tissue homogenization were 7.5–15 mg total per replicate. Each sample was homogenized for 30 seconds at full speed, and immediately centrifuged. DNA and RNA were dually extracted from each aliquot following the manufacturer’s instructions, including the DNase digestion step during RNA purification. RNA was eluted in a final volume of 40 μl; DNA was eluted in a final volume of 100 μl.

### Library preparation and sequencing for Whole Exome and Whole Transcriptome Sequencing

Whole Exome Sequencing: Extracted DNA samples were quantified using the Qubit 1x dsDNA HS Assay. The DIN of each sample was measured using the TapeStation 4200 and Genomic DNA ScreenTape to determine the input as 100ng (Agilent). Samples were normalized to 5 ng/μL with 1x Low EDTA TE buffer. If concentration of a sample was lower than 5 ng/μL, a speed vacuum method was used to concentrate sample to 5 ng/μL. Nuclease-free water was used as non-template control (NTC) and HD753 was used as the positive control. DNA-seq libraries were prepared and sequenced following the SureSelect XT HS2 DNA System Protocol (SureSelect XT HS2 DNA Kits-Library Preparation (+/−MBC) / Fast-Hyb Target Enrichment/Post-capture Pooling Workflow For Illumina Platform NGS (Version F0, May 2023), SureSelect XT HS2 DNA System (Version E0, July 2022)).

Whole Transcriptome Sequencing: Extracted RNA samples were quantified using the Qubit RNA HS Assay. The DV200 of each sample was measured using the TapeStation 4200 and High Sensitivity RNA ScreenTape to determine the input amount as 50ng (Agilent). Samples were normalized using nucleasefree water to 5 ng/μL. If concentration of sample was lower than 5 ng/μL, a speed vacuum method was used to concentrate sample to 5 ng/μL. Nuclease-free water was used as the non-template control (NTC) and RNA extracted from Seraseq FFPE Fusion RNA Reference Material v4 was used as the positive control. RNA-seq libraries were prepared and sequenced following SureSelect XT HS2 RNA System Protocol (SureSelect XT HS2 RNA System Protocol (Version C0, June 2022)).

### Bioinformatics Analysis for Whole Exome and Whole Transcriptome Sequencing

Whole Exome Sequencing: Prior to variant discovery, the raw sequencing data was pre-processed using Agilent Genomics NextGen Toolkit (AGeNT) (Agilent, Version 3.1). First, sequencing adaptors were removed and molecular barcode information extracted using the AGeNT Trimmer tool. Next, the reads were aligned to human genome reference build GRCh37 using the *BWA-MEM* aligner^65^. Lastly, analy-sisready reads aligned-BAM files were generated using the AGeNT CReaK tool that performs PCR de-duplication leveraging the molecular barcode information. Alignment quality was evaluated using QualiMap^66^, which provides metrics including read alignment distribution, mapping quality, insert size and coverage uniformity. Variant calling was performed using Illumina Pisces, a somatic small-variant calling application^67^.

Whole Transcriptome Sequencing: The FASTQ files with raw reads were aligned to the human genome using reference build GRCh37 and the corresponding GENCODE annotation version 19. Quality control for the raw reads was performed with *FASTQC* software^68^. Spliced alignment of the reads to the reference genome was done with *STAR* software^69^, allowing a maximum of 1 mismatch per read and its quality control was done using *RSeQC* software^70^. Mapped reads were quantified at the gene level in a raw counts matrix using *Subread* software^71,72^ using fracOverlap 1 (only entire reads overlapping to annotation feature are counted).

### DNA Extraction for Nanopore Sequencing

DNA was extracted from 50–200uL of cryopreserved aspirate or sPT sample via the ZymoBIOMICS MagBead DNA/RNA kit (Zymo Research) following manufacturer’s instructions, except for the final elution, which was conducted in 200 uL of DNAse/RNAse Free Water and incubated at 37C for 10 minutes to recover long fragments. To achieve an ideal fragment length distribution for nanopore sequencing, DNA was then sheared using a 26G 1” needle for 7 passes and subsequently size selected using 0.4x volumes of Ampure XP Beads (Beckman Coulter). DNA was quantified using a Quantus fluorometer with the Qubit dsDNA Quantification, High Sensitivity Assay Kit (ThermoFisher Scientific).

### Nanopore Sequencing

Ligation-based library preparation of native genomic DNA was conducted with either the SQK-LSK114 or SQK-NBD114 kits. For the SQK-LSK114 kit, all library preparation was conducted following the manufacturer’s instructions, with the exception of replacing the recently recommended Salt T4 Ligase with the previously recommended Quick T4 Ligase. The manufacturer’s optional 37C incubation for 10 minutes in the final elution was also used to recover long fragments. For the SQK-NBD114 kit, all library preparation was conducted following the manufacturer’s instructions, with the exception of pooling barcoded samples after, rather than before, the post-barcode ligation clean-up step. Samples were sequenced with either one (SQK-LSK114) or two (SQK-NBD114) samples per FLO-PRO114M flow cell, for a duration of either 72 hours or until all pores were exhausted. All samples were sequenced on a PromethION 2 Solo (P2) sequencer, live basecalled using Dorado (v0.8.3) super accurate mode with 5hmCG and 5mCG modification calling (basecall model: dna_r10.4.1_e8.2_400bps_sup@v4.3.0), and de-multiplexed, if applicable. Basecalled reads were aligned to the T2T/chm13v2.0 human reference genome assembly using Dorado aligner.

### Methylation Analysis for Nanopore Sequencing

As recommended by Sturgeon^56^, the aligned bam file(s) output by Dorado were then sorted before being input into modkit adjust-mods --convert h m^73^, which combines 5-methylcytosine and 5-hydroxymethylcytosine calls into a single methylation probability for each CG site of each read. The large table produced by modkit convert was then piped into modkit extract full, a function that takes a given bed file and extracts only methylation probabilities within the given 50bp range of each of the 427,859 CG sites used by Sturgeon. The output of modkit extract full was then piped into an in-house version of Sturgeon inputtobed that best recapitulates the data wrangling completed in the original publication.

Briefly, this in-house function averages all methylation probabilities across reads on a per-site basis and then discretizes the probability to 0 if average is < 0.6 and to 1 if average is ≥ 0.6, a common practice in the field. The resulting per-site methylation probabilities were then used as input to Sturgeon predict for methylation-based. The t-SNE was created using the Rtsne package. To perform inter-patient methylation correlations, we identified 378,673 sites consistently used in methylation classification across all matched pairs. We used a validated probe set^56^ and identified 345,518 CG confidently covered sites used for classification for the nanopore samples. We identified the 20,221 top variable CG sites of the data set^56^ (SD>0.228) and applied to the nanopore sequenced samples, leading to a total of 16,124 CG sites used for high-dimensional visualization.

#### Functional Validation of Aspirate Tissue

Aspirate samples were cultured under four media conditions: brain slice media (BSM), serum-free neurobasal media with EGF and FGF (EF), neurobasal-based media from the Ian’s Friends Foundation (IFF), and DMEM with 10% fetal bovine serum (FBS). Viability was assessed on day 4 via bioluminescent imaging, both in vitro and on OBSCs.

Matched aspirate and standard pathology tissue (sPT) samples from three patients were tested using 337 drug–tumor combinations. For direct comparisons, 15 combinations were analyzed using unpaired t-tests in GraphPad Prism.

#### Combination Therapy Testing

Combination regimens were selected by clinicians and administered on day 1 of the SLiCE assay. Radiation was delivered prior to drug exposure when applicable. Viability was measured on day 4 using bioluminescent imaging.

Synergy was quantified using SynergyFinder and the Highest Single Agent (HSA) model. Synergy scores were categorized as antagonistic (< −10), additive (−10 to 10), or synergistic (> 10).

#### Quality Control and Reproducibility

Cryopreserved aspirate tissue was compared to fresh samples using four chemotherapeutics and analyzed via two-way ANOVA. Aliquot reproducibility was assessed by parallel testing of multiple cryopreserved aliquots from the same patient. Longitudinal stability was evaluated across three time points using aspirate from a single patient.

OBSC health was monitored using propidium iodide staining. A viability threshold of ≥80% was preferred, with a minimum of 55%. Z′ factor analysis was performed for each OBSC run.

#### Statistical Analysis

All analyses were performed using GraphPad Prism v10.5.0. One-way ANOVA with Tukey’s multiple comparisons test was used for multi-group comparisons. Unpaired t-tests were used for two-group comparisons. Two-way ANOVA was applied for dose and tissue source comparisons.

ROC curves and AUC values were generated to assess predictive performance. Hazard ratios were calculated using log-rank tests. Fisher’s exact test was used for contingency analyses.

Statistical significance was defined as follows:

- *p* < 0.0332 (*),
- *p* < 0.0021 (**),
- *p* < 0.0002 (***),
- *p* < 0.0001 (****)

For two-way ANOVA, thresholds were:

- *p* < 0.05 (*),
- *p* < 0.01 (**),
- *p* < 0.001 (***),
- *p* < 0.0001 (****).

## Supplementary Material

Supplementary Files

This is a list of supplementary files associated with this preprint. Click to download.

- MannetalSupplementalInfo.pdf

## Data Availability

Any information required to reanalyze the data reported in this paper is available from the lead contact upon request.
